# Heterogeneous effects of genetic risk for Alzheimer’s disease on the phenome

**DOI:** 10.1038/s41398-021-01518-0

**Published:** 2021-07-23

**Authors:** Hei Man Wu, Alison M. Goate, Paul F. O’Reilly

**Affiliations:** 1 Department of Genetics and Genomic Sciences, Icahn School of Medicine, Mount Sinai, New York, NY USA; 2 Department of Neurosciences, Icahn School of Medicine at Mount Sinai, New York, NY USA

**Keywords:** Genetics, Personalized medicine

## Abstract

Here we report how four major forms of Alzheimer’s disease (AD) genetic risk—APOE-ε4, APOE-ε2, polygenic risk and familial risk—are associated with 273 traits in ~500,000 individuals in the UK Biobank. The traits cover blood biochemistry and cell traits, metabolic and general health, psychosocial health, and cognitive function. The difference in the profile of traits associated with the different forms of AD risk is striking and may contribute to heterogenous presentation of the disease. However, we also identify traits significantly associated with multiple forms of AD genetic risk, as well as traits showing significant changes across ages in those at high risk of AD, which may point to their potential roles in AD etiology. Finally, we highlight how survivor effects, in particular those relating to shared risks of cardiovascular disease and AD, can generate associations that may mislead interpretation in epidemiological AD studies. The UK Biobank provides a unique opportunity to powerfully compare the effects of different forms of AD genetic risk on the phenome in the same cohort.

## Background

Genetic risk for Alzheimer’s disease (AD) takes several forms, including: Mendelian, rare, polygenic, APOE and family risk [1]. It is important to investigate the impact that these different forms of AD risk have on the array of biomarkers, traits and clinical outcomes comprising the ‘phenome’ for two major reasons. (1) Heterogeneity in the presentation and pathophysiology of AD has been widely reported [2–4]. A part of this heterogeneity may be caused by the different effects that different risk factors have on the phenome. (2) Growing evidence suggests that modifiable factors, such as exercise, diet and alcohol consumption, contribute substantially to the risk of late-onset Alzheimer’s Disease (LOAD) [5, 6]. Any modifiable risk factor, or active biological compound on which it acts, will have a genetic component. This genetic component must also be a genetic component of AD itself, since it initiates a path towards a higher risk of AD. Therefore, one strategy for identifying candidate modifiable risk factors is to investigate the downstream effects of genetic risk for AD [6]. While such an approach will highlight many traits due to (horizontal) pleiotropic effects on the trait and AD, it will produce a long list of mediation candidates. In combination with evidence from other sources, these candidates can then be prioritized for further study, which may ultimately lead to the identification of targets for intervention.

The APOE gene contains risk alleles for LOAD that are among the largest common genetic effects on any disease, with homozygous APOE-ε4 carriers ~12 times more likely to develop the disease [7, 8]. While the precise mechanism of APOE-ε4’s role in the pathogenesis of AD is unknown, APOE has reported roles in B-amyloid clearance, lipid homeostasis and cholesterol transport [7, 9]. In contrast, APOE-ε2 has apparent protective effects against AD. As well as reduced incidence of LOAD, APOE-ε2 is associated with increased cognitive reserve [10, 11], reduced cortical thinning and lower amyloid-ß accumulation [11]. Outside the APOE gene, the genetic effects on LOAD are thought to be largely polygenic: 21 significant loci were identified in the latest GWAS of LOAD [12], while the estimated *h*_SNP_^2^ is 7.1%, and after removal of the APOE region is 6.4% [12]. Polygenic Risk Scores (PRS), which combine the effects of many risk alleles genome-wide to provide a proxy of polygenic risk to a disease [13], have been widely used in AD research, finding associations with AD clinical diagnosis [14, 15], disease progression [16], cognitive impairment [15, 16], and educational attainment in children [17]. An alternative proxy for the genetic risk of AD is family history of AD, which reflects both common and rare genetic risk variants, risk variants for early and late onset AD, as well as environmental risks. In recent years, GWAS performed on family history of AD [18] or “AD-by-proxy” [19], based on parental AD/dementia status and age, has been demonstrated as a powerful way of identifying risk variants for AD.

In this study, we report the associations between AD genetic risk and a range of traits in the UK Biobank, some of which may mediate risk for AD. We examine four major forms of AD genetic risk: (1) APOE-ε4, the strongest known genetic factor for LOAD, which is also associated with early-onset AD [20], (2) APOE-ε2, a protective allele of AD, (3) Polygenic risk, using the AD PRS (with APOE excluded) as a proxy, and (4) Familial risk, according to the parental history of AD. A key advantage of this study is the unique opportunity that the UK Biobank provides to systematically and powerfully compare the effects of these four forms of genetic risk across a large number of traits, in the same cohort. We utilize the rich data resources of the UK Biobank to investigate hundreds of traits relating to education, cognitive function, lifestyle and environment, psychosocial factors, health and medical history, and physical and biological measures. Our findings highlight substantial heterogeneity in the profile of traits associated with different forms of AD genetic risk and the phenome.

## Methods

### Study population

This study used the UK Biobank (UKB) data [21], which is a large prospective multi-ancestry cohort study of ~500,000 participants (European ancestry samples, *n* = 472,725; non-European ancestry samples, *n* = 27,034), aged 40–69 years when baseline measures were taken during recruitment across the UK between 2006 and 2010. We used the genotype and phenotype data generated from the UKB.

Dementia cases including AD, vascular dementia, frontotemporal dementia and all-cause dementia were removed from analyses to limit the downstream effects of the disease on observed trait values. Cases were identified as having a primary/secondary diagnosis from hospital records or primary/secondary cause of death from the death registry, using ICD-10 codes in categories F01-F99 (mental and behavioural disorder chapter) or G00-G99 (disease of the nervous system chapter). Participants who self-reported having dementia/Alzheimer’s/cognitive impairment (F20002) were also excluded. A total of 1532 dementia cases were identified and excluded from the dataset.

### Genetic risk factors

#### APOE risk

APOE contains three common forms of the “ε allele” associated with AD risk: ε2, ε3 and ε4. The ε4 allele is the strongest common genetic risk factor for LOAD, while ε2 is associated with substantially reduced risk relative to the ε3 allele, which is typically considered the reference allele in association testing. Which of the three APOE ε alleles (or haplotypes) an individual has on each chromosome is defined by their alleles at two missense SNPs, rs7412 and rs429358, and APOE ε genotypes are the pair of these an individual has on their two chromosomal strands (e.g. ε2/ε4, ε3/ε3, ε3/ε4). The two SNPs defining the ε alleles were directly genotyped in the UK Biobank (Hardy-Weinberg *P* > 0.05; genotyping rate > 80%), but do not pass standard GWAS quality control (QC), which typically requires genotyping rate > 95%. However, in this study, these two SNPs were retained to define the APOE ε genotype given their known importance to AD risk. The concordance between the genotype calls of the two SNPs from the genotyping array and from whole-exome sequencing among 34,453 individuals of the first phase of whole-exome sequencing in the UK Biobank is extremely high (rs7412: 99.93%; rs429358: 99.96%).

We investigated the dosage effects of the ε4 and ε2 alleles relative to the ε3 allele. Specifically, to test the effect of APOE-ε4 on traits we considered an additive genetic model with ε3/ε3 (*n* = 242,205) coded as 0, ε3/ε4 heterozygotes (*n* = 97,137) as 1, and ε4/ε4 (n = 9,693) coded as 2. To test the effect of APOE-ε2 on traits we coded ε3/ε3 as 0, ε3/ε2 heterozygotes (n = 501,99) as 1, and ε2/ε2 homozygotes (*n* = 2,329) as 2. In common with other AD studies [22, 23], participants with ε2/ε4 were excluded to avoid their effects being conflated. A total of 401,563 individuals with complete genotype data at rs7412 and rs429358 to determine APOE alleles were available for analysis.

#### Polygenic risk

PRSice-2 [24] was used to construct polygenic risk scores (PRS) using the latest GWAS of LOAD [12] (*n* = 94,437) as base data, and the UK Biobank sample as target data. Due to restricted permissions associated with using the latest GWAS of LOAD [12] to predict education and intelligence outcomes, an earlier GWAS of LOAD [25] was used as base data for predicting traits in the Cognitive function category. In our analyses, we selected genotyped SNPs (*n* = 560,173) with minor allele frequency > 1%, Hardy-Weinberg Equilibrium test *P*-value > 1 × 10^−8^ and genotyping rate >98%. Genetic relatedness analyses were performed using KING [26], and we discarded one of each pair of individuals with up to 3rd-degree relationships (KING *r*^2^ > 0.044). Note that only individuals of European ancestry were included in this part of the analysis because the base GWAS data [12, 25] were performed in European ancestry individuals, meaning that systematic differences and misleading results may be generated due to the poor generalisability of PRS across ancestry samples using present PRS methods [13]. Individuals of European ancestry were identified using 4-means clustering on the first two principal components provided by the UKB and were retained in the analyses. SNPs residing within 1 Mb of the APOE gene (chr19: 44409039-46412650; hg19 assembly) were excluded in the PRS calculation to capture genetic signals outside the major risk gene.

In order to select the optimal *P*-value threshold for PRS calculation, we used the “AD-by-proxy” approach that has been successfully used to perform “GWAX” analyses in AD [19]. In brief, AD-by-proxy is a proxy-phenotype, which is computed for each individual based on the age and AD statuses of their parents. Following [19], the proxy AD phenotype was calculated as the count of the number of affected biological parents. For those with unaffected parents, the contribution to the count was weighted by their parents’ current ages (or age at death, if applicable; see [19] for details). Individuals with missing data on parental age or AD status were removed. PRS in 384,635 individuals were calculated using 117 SNPs after identifying the optimal *P*-value threshold (*P*-value < 0.0001) for predicting AD-by-proxy status in 337,336 individuals (*P*-value = 6.2 × 10^−53^). Among the 117 SNPs, the nearest SNPs to the APOE gene are ~7 Mb (rs12459419) and 14 Mb (rs7258465) away and, thus, the effects of the PRS should be independent of APOE effects.

#### Familial risk

Family history of AD was determined by self-report. Participants were asked whether their father/mother ever suffered from AD/dementia (F20110, F20107). Data from the initial assessment visit (2006–2010), the report assessment visit (2012–2013) and the imaging visit (2014+) were aggregated. Individuals with maternal and/or paternal AD are considered to have a positive family history of AD. Since there is a limited number of samples whose siblings were affected by AD (*n* = 3227), and these individuals may differ systematically from those with parents with AD, we did not include siblings in the definition of familial risk. To evaluate familial risk separately from the heritable genetic effect of APOE, we controlled for the APOE genotypes when using familial risk as a genetic risk factor. Among the sample with information on APOE genotype, 319,597 individuals have no parents with reported AD/dementia (coded as 0), 41,490 individuals have one parent with AD/dementia (coded as 1), and 1574 individuals have both parents with AD/dementia (coded as 2).

### Traits

Data on a total of 273 traits from the UK Biobank across six broad categories—blood biochemistry (e.g. CRP, cholesterol, Vitamin D), blood cell traits (e.g. RBC, Platelet dist. width), metabolic health (e.g. BMI, Diabetes, dietary intake), general health (e.g. lung function, allergies), psychosocial factors (e.g. depression, family satisfaction), cognitive function (e.g. education, trail-making-test)—were included in this study (see Supplementary Table 1 for details of all traits). These putative risk factors have been extensively reported in the study of potential candidates of intervention of dementia/AD [6, 27–29]. Here we include a wider and higher-resolution collection of traits falling into the broad categories of these known risk factors, available in the UK Biobank. Supplementary Note 1 contains details about data processing and coding. For traits with 10 or more unique values, we removed outliers >6 standard deviations from the mean and applied a rank-based inverse normal transformation to all.

### Statistical analysis

We first tested for associations between the AD genetic risk factors and all 273 traits, using linear and logistic regression for continuous and binary traits, respectively. All models were adjusted by age, age^2^, sex, an age*sex interaction term, socioeconomic status based on Townsend deprivation index, assessment centre, and, for the genetic analyses, genotyping batch and the first 12 principal components of ancestry. We additionally adjusted for APOE genotype when assessing familial risk as a genetic risk factor, to account for the effect of APOE in familial risk. Models for which the blood biochemistry traits were outcomes were also adjusted for fasting time and dilution factor.

We next conducted analyses that additionally adjusted for paternal and maternal ages in all models. These were performed to account for the potential health benefits conferred in genetics derived from parents with high longevity. We used the parents’ age at death (Mother’s age at death: F3526; Father’s age at death: F1807) if applicable. For participants who reported their father or mother still alive at baseline, we estimated their parent’s life expectancy given mother’s or father’s age and the year of baseline assessment, using data from the Office for National Statistics (https://www.ons.gov.uk/).

To investigate potential confounding by cardiovascular disease (CVD), stratified analyses were performed by grouping the individuals into cases and controls in each of the following categories: statin use (F20003), self-reported CVD (i.e. heart attack, angina, stroke or high blood pressure: F6150), and having a biological parent with high blood pressure, stroke, or heart disease (F20107, F20110).

**Fig. 1:**
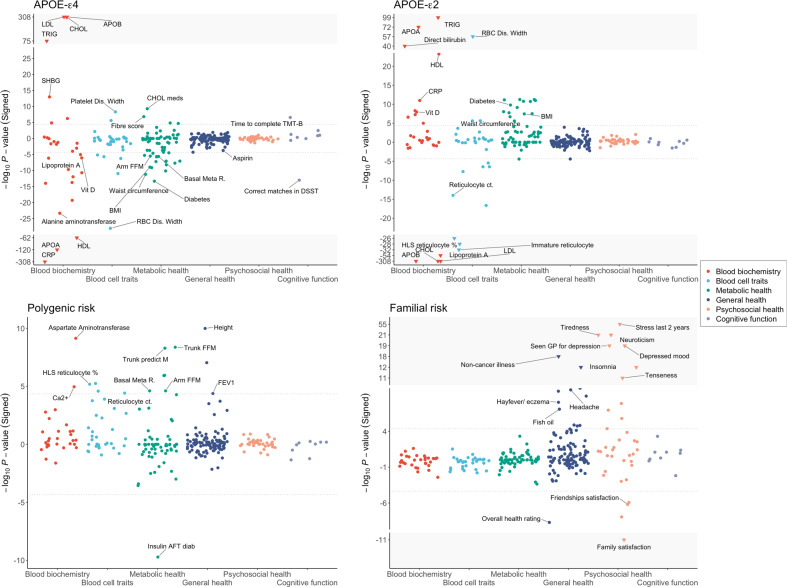
Associations between APOE-ε4, APOE-ε2, polygenic risk (AD PRS) and AD familial risk and 273 traits in the UK Biobank. These association analyses between the genetic risk factors and UK Biobank traits (described in the “Methods” section) were controlled for age, age^2^, sex, age*sex interaction term, socioeconomic status based on Townsend deprivation index, genotyping batch, assessment centre, and the first 12 principal components of ancestry, self-reported CVD, statin use, parental CVD and lifestyle score in the models. The grey dotted lines correspond to the *P*-value significance threshold (*P* < 4.5 × 10^−5^; see Methods). The *Y*-axis has been truncated to show all associated traits to improve visualization, with extreme results shown in the grey area with inverted triangles.

Given that the CVD stratified analyses showed a substantial difference in APOE-ε4 effects between CVD cases and controls for a subset of traits, in particular metabolic health traits, in our primary analyses (Fig. 1) we additionally included adjusting for these factors (i.e. statin use, self-reported CVD condition, family history of CVD), and a healthy lifestyle score, as covariates. Briefly, the healthy lifestyle score [28] is a composite score consisting of smoking, physical activity, diet and alcohol consumption. The purpose of adjusting for the healthy lifestyle score was to account for the potential ascertainment of healthier lifestyle and genetics among living individuals of middle or older age with higher risk for CVD due to APOE-ε4.

A conservative Bonferroni correction, assuming independence of all tests, was used to control for multiple testing in the analyses described above. A P-value threshold of 4.5 × 10^−5^ was used (0.05/273(number of traits)*4(number of predictors)) for inferring statistical significance. The correlation between the sets of results among the four forms of AD genetic risk is assessed by Kendall’s rank correlation test, in each of the six trait categories separately. A *P*-value threshold of 0.00139 was used (0.05/6(number of categories)*6(number of pairwise correlations among the AD predictors)) for inferring significance. For the 98 traits with significant associations (*P* < 4.5 × 10^−5^) in the final model, we then tested for interactions between the AD genetic risk factor and age. To investigate the sex-specific genetic effects, we also performed the analysis stratified by sex and tested for differences between their results using *t*-tests. A *P*-value threshold of 6.4 × 10^−5^ was used (0.05/98(number of traits)*4(number of predicators)*2(age and sex interaction tests)) for inferring significance.

## Results

In this study, we examined associations between four forms of AD genetic risk and 273 traits across the UK Biobank. Standard linear and logistic regression analyses were performed, controlling for covariates including age, sex, genetic principal components, parental age, CVD factors and lifestyle score (see Methods). Parental age, CVD factors and lifestyle score were only added to our model after observing the effects of potential confounding in our initial analyses (see below).

These analyses identified significant associations (*P* < 4.5 × 10^−5^) between APOE-ε4 and 51 traits, APOE-ε2 and 42 traits, polygenic risk and 16 traits, and familial risk and 27 traits (Fig. 1, Table 1, Supplementary Table 1). While the four forms of AD genetic risk inevitably have a different power to identify significant associations with the traits, each is sufficiently powered to detect multiple traits and the broad profile of associations is noticeably different across the four forms. APOE-ε4 dosage is primarily associated with blood biochemistry traits, metabolic health and cognitive function, while APOE-ε2 dosage shows the opposite associations for most of these traits, although not with cognitive function. Both polygenic (excluding APOE) and familial risk (controlling for APOE genotype) provide proxies for genome-wide genetic risk outside the APOE region. While AD PRS is associated with a range of traits across the categories of blood biochemistry, blood cell traits, metabolic health and general health, familial risk for AD is primarily associated with psychosocial health.

**Table 1:** Details of the 30 most significant associations between APOE-ε4, APOE-ε2, polygenic risk (AD PRS) and AD familial risk and traits in the UK Biobank.

Phenotype	Category	APOE-ε4			APOE-ε2			Familial risk			Polygenic score		
		Est (SE)	*P*	*r*^2^	Est (SE)	*P*	*r*^2^	*Est (SE)*	*P*	*r*^2^	Est (SE)	*P*	*r*^2^
Apolipoprotein B	Blood biochemistry	0.24 (0.0039)	**<1** **×** **10**^**−308**^	0.025	−0.69 (0.0055)	**<1** **×** **10**^**−308**^	0.09	0.007 (0.0064)	0.28	9.5 × 10^−4^	−0.2 (0.82)	0.8	2.9 × 10^−7^
C reactive protein	Blood biochemistry	−0.26 (0.0041)	**<1** **×** **10**^**−308**^	0.02	0.039 (0.0057)	**1.1** **×** **10**^**−11**^	0.0023	5.2 × 10^−4^ (0.0068)	0.94	−9.8 × 10^−5^	1.2 (0.83)	0.14	3.3 × 10^−5^
Cholesterol	Blood biochemistry	0.17 (0.0039)	**<1** **×** **10**^**−308**^	0.013	−0.42 (0.0055)	**<1** **×** **10**^**−308**^	0.036	0.0075 (0.0065)	0.25	5.3 × 10^−4^	0.048 (0.8)	0.95	1.1 × 10^−5^
LDL direct	Blood biochemistry	0.19 (0.0039)	**<1** **×** **10**^**−308**^	0.015	−0.54 (0.0055)	**<1** **×** **10**^**−308**^	0.058	0.0046 (0.0065)	0.48	9.1 × 10^−4^	−0.089 (0.82)	0.91	5.4 × 10^−8^
Apolipoprotein A	Blood biochemistry	−0.092 (0.0039)	**5.4** **×** **10**^**−121**^	0.0021	0.099 (0.0055)	**4.6** **×** **10**^**−73**^	5.8 × 10^−4^	−3.2 × 10^−4^ (0.0065)	0.96	−8.4 × 10^−4^	0.57 (0.79)	0.47	2.6 × 10^−6^
Triglycerides	Blood biochemistry	0.073 (0.004)	**2.4** **×** **10**^**−75**^	−0.001	0.12 (0.0056)	**2.4** **×** **10**^**−99**^	0.0022	0.0039 (0.0066)	0.55	7.1 × 10^−4^	0.62 (0.8)	0.44	2.8 × 10^−6^
HDL cholesterol	Blood biochemistry	−0.064 (0.0038)	**5.5** **×** **10**^**−63**^	9.4 × 10^−4^	0.054 (0.0053)	**6.1** **×** **10**^**−24**^	−0.0012	0.0027 (0.0063)	0.66	−0.001	0.51 (0.77)	0.51	2.0 × 10^−6^
RDW	Blood cell traits	−0.05 (0.0045)	**8.5** **×** **10**^**−29**^	5.8 × 10^−4^	0.1 (0.0063)	**1.0** **×** **10**^**−57**^	0.0023	−0.0011 (0.0074)	0.88	−3.8 × 10^−5^	−0.48 (0.88)	0.59	1.4 × 10^−6^
Illness, injury...*	Psychosocial health	−0.015 (0.0086)	0.093	−0.0011	0.005 (0.012)	0.68	9.0 × 10^−5^	0.22 (0.014)	**1.4** **×** **10**^**−55**^	0.0012	−0.51 (1.7)	0.77	2.5 × 10^−7^
Lipoprotein A	Blood biochemistry	−0.024 (0.0048)	6.9 × 10^−7^	4.1 × 10^−4^	−0.11 (0.0068)	**2.5** **×** **10**^**−54**^	0.001	−0.0081 (0.008)	0.31	−3.1 × 10^−4^	−1.9 (0.98)	0.053	2.1 × 10^−5^
Direct bilirubin	Blood biochemistry	−0.029 (0.0044)	2.0 × 10^−11^	0.0018	0.081 (0.006)	**1.1** **×** **10**^**−40**^	0.0015	−0.017 (0.0072)	0.022	5.3 × 10^−4^	0.11 (0.87)	0.9	5.9 × 10^−7^
Immature retic. fraction	Blood cell traits	−0.023 (0.0046)	5.6 × 10^−7^	5.1 × 10^−5^	−0.076 (0.0064)	**1.7** **×** **10**^**−32**^	9.9 × 10^−4^	0.0048 (0.0076)	0.52	3.7 × 10^−5^	3 (0.9)	8.1 × 10^−4^	1.5 × 10^−4^
HL retic. percentage	Blood cell traits	−0.0066 (0.0046)	0.15	−3.2 × 10^−6^	−0.071 (0.0064)	**1.1** **×** **10**^**−28**^	8.5 × 10^−4^	0.0023 (0.0075)	0.76	2.2 × 10^−5^	4.1 (0.9)	**6.6** **×** **10**^**−6**^	1.0 × 10^−4^
HL retic. count	Blood cell traits	−0.0072 (0.0046)	0.12	8.5 × 10^−5^	−0.069 (0.0064)	**9.1** **×** **10**^**−27**^	7.9 × 10^−4^	0.0018 (0.0076)	0.81	4.8 × 10^−5^	4.1 (0.9)	**5.7** **×** **10**^**−6**^	1.0 × 10^−4^
Alanine aminotransferase	Blood biochemistry	−0.04 (0.0039)	**4.3** **×** **10**^**−24**^	−7.8 × 10^−4^	0.018 (0.0055)	0.0014	−4.4 × 10^−4^	0.0066 (0.0065)	0.31	0.0011	0.79 (0.79)	0.32	1.1 × 10^−6^
Neuroticism score	Psychosocial health	0.0042 (0.014)	0.77	2.3 × 10^−4^	0.045 (0.02)	0.028	4.7 × 10^−4^	0.23 (0.024)	**9.3** **×** **10**^**−22**^	5.3 × 10^−4^	0.078 (2.9)	0.98	3.8 × 10^−9^
Freq. of tiredness...*	Psychosocial health	−0.0067 (0.0033)	0.041	4.9 × 10^−4^	0.011 (0.0046)	0.016	6.0 × 10^−4^	0.051 (0.0054)	**1.6** **×** **10**^**−21**^	−5.4 × 10^−06^	0.35 (0.65)	0.59	1.3 × 10^−6^
Glycated haemoglobin	Blood biochemistry	−0.037 (0.0041)	**4.7** **×** **10**^**−20**^	−5.1 × 10^−4^	0.011 (0.0057)	0.059	0.0023	−0.0093 (0.0067)	0.17	3.5 × 10^−4^	−0.76 (0.81)	0.35	4.2 × 10^−6^
Seen GP for nerves...*	Psychosocial health	0.004 (0.0092)	0.66	5.5 × 10^−4^	0.0042 (0.013)	0.74	−2.0 × 10^−4^	0.14 (0.015)	**7.5** **×** **10**^**−20**^	5.0 × 10^−4^	0.57 (1.8)	0.76	3.4 × 10^−7^
Freq. of depressed...*	Psychosocial health	0.0027 (0.0024)	0.27	−4.0 × 10^−5^	0.0022 (0.0034)	0.52	4.3 × 10^−4^	0.036 (0.004)	**2.5** **×** **10**^**−19**^	1.8 × 10^−5^	0.19 (0.48)	0.7	1.5 × 10^−5^
Non-cancer illnesses...*	General health	−0.0017 (0.0032)	0.6	0.0059	0.011 (0.0044)	0.013	−0.0053	0.046 (0.0052)	**9.4** **×** **10**^**−19**^	−0.0027	0.074 (0.64)	0.91	1.4 × 10^−6^
Retic. percentage	Blood cell traits	0.0039 (0.0046)	0.4	1.2 × 10^−4^	−0.055 (0.0064)	**2.3** **×** **10**^**−17**^	5.0 × 10^−4^	5.0 × 10^−4^ (0.0076)	0.95	2.3 × 10^−5^	3.8 (0.9)	**2.6** **×** **10**^**−5**^	1.4 × 10^−4^
Creatinine	Blood biochemistry	−0.026 (0.0034)	**9.9** **×** **10**^**−15**^	0.0013	0.024 (0.0047)	**2.5** **×** **10**^**−7**^	6.2 × 10^−4^	−0.0084 (0.0056)	0.13	4.9 × 10^−4^	1.2 (0.68)	0.085	1.4 × 10^−5^
Retic. count	Blood cell traits	0.0037 (0.0046)	0.42	2.2 × 10^−4^	−0.05 (0.0064)	**1.0** **×** **10**^**−14**^	4.3 × 10^−4^	3.0 × 10^−4^ (0.0076)	0.97	4.8 × 10^−5^	3.7 (0.9)	**3.9** **×** **10**^**−5**^	1.4 × 10^−4^
Alkaline phosphatase	Blood biochemistry	−0.031 (0.0041)	**1.7** **×** **10**^**−14**^	2.0×10^−4^	0.025 (0.0057)	**1.0** **×** **10**^**−5**^	8.5 × 10^−4^	−0.0061 (0.0067)	0.36	8.7 × 10^−4^	2.2 (0.82)	0.0061	1.3 × 10^−5^
Diabetes*	Metabolic health	−0.18 (0.024)	**4.5** **×** **10**^**−14**^	0.0016	0.21 (0.032)	**1.6** **×** **10**^**−10**^	0.0074	−0.062 (0.039)	0.11	0.0018	−14 (4.7)	0.0038	1.6 × 10^−4^
Substitution Test*	Cognitive function	−0.053 (0.0071)	**9.5** **×** **10**^**−14**^	−2.7 × 10^−4^	−0.0066 (0.0098)	0.5	−0.0058	−0.03 (0.011)	0.0065	−0.0011	−1.1 (1.2)	0.32	1.6 × 10^−5^
SHBG	Blood biochemistry	0.028 (0.0038)	**1.1** **×** **10**^**−13**^	0.0019	0.0085 (0.0054)	0.11	−0.0056	−0.011 (0.0063)	0.082	0.0014	−0.5 (0.77)	0.51	2.2 × 10^−6^
Insomnia	General health	−0.0094 (0.003)	0.0016	1.0 × 10^−4^	0.009 (0.0042)	0.03	7.5 × 10^−4^	0.035 (0.0049)	**5.2** **×** **10**^**−13**^	2.9 × 10^−4^	−0.066 (0.6)	0.91	1.3 × 10^−7^
Urea	Blood biochemistry	−0.029 (0.004)	**1.0** **×** **10**^**−12**^	9.6 × 10^−5^	0.011 (0.0056)	0.043	7.9 × 10^−4^	−0.0055 (0.0066)	0.4	1.6 × 10^−4^	1.4 (0.81)	0.078	1.2 × 10^−6^

These analyses correspond to the 30 most significant associations from Fig. 1, in which association analyses between the genetic risk factors and UK Biobank traits (described in the “Methods” section) were controlled for age, age^2^, sex, age*sex interaction term, socioeconomic status based on Townsend deprivation index, genotyping batch, assessment centre, and the first 12 principal components of ancestry, self-reported CVD, statin use, parental CVD and lifestyle score in the models. *P*-values shown in bold a significant (*P* < 4.5 × 10^−5^; see Methods). See Supplementary Table 1 for full association results corresponding to all 273 traits.

*RDW* Red blood cell erythrocyte distribution width; Illness injury…* Illness, injury, bereavement, stress in last 2 years; retic. reticulocyte; *HL* High light scatter; Freq. of tiredness…* Frequency of tiredness/lethargy in last 2 weeks; Seen GP for nerves…* Seen doctor (GP) for nerves, anxiety, tension or depression; Freq. of depressed…* Frequency of depressed mood in last 2 weeks; Non-cancer illnesses* Number of self-reported non-cancer illnesses; Diabetes* Diabetes diagnosed by doctor; Substitution Test* Symbol Digit Substitution Test.

In order to provide context to these results, we performed the PRS and familial risk analyses in relation to Parkinson’s disease, major depressive disorder, diabetes, and height, each of which showed a markedly different profile of associations with the 273 traits tested (Supplementary Figs. 1 and 2), highlighting the specificity of the AD results and providing reassurance that they are not due to artifact or cohort effects.

**Fig. 2:**
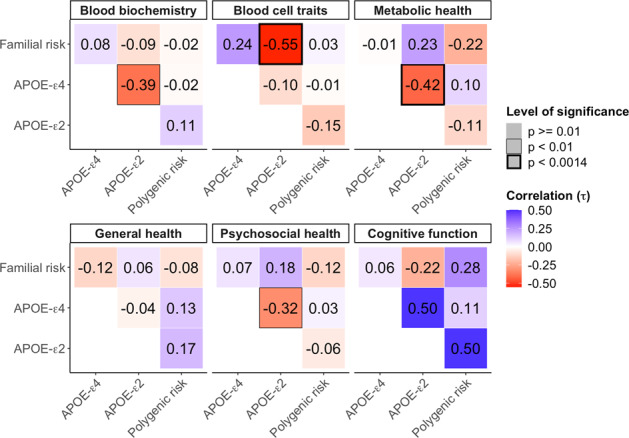
Correlations between the trait association results of different forms of AD genetic risk. The value reported in each cell is the rank correlation coefficient (Kendall’s τ) between the genetic-trait association results, illustrated in Fig. 1, of the corresponding forms of genetic risk. All the results are adjusted for age, age^2^, sex, age*sex interaction term, socioeconomic status based on Townsend deprivation index, genotyping batch, assessment centre, and the first 12 principal components of ancestry, self-reported CVD, statin use, parental CVD and lifestyle score (see Methods). Correlations of nominal significance (*P* < 0.01) are highlighted by a box, while those that are significant after correction for multiple testing (*P* < 0.0014; see Methods) are highlighted by a bold box.

We examined the rank correlation between the results of Fig. 1 generated by the different forms of AD genetic risk (Fig. 2) to quantify the level of convergence or divergence of AD genetic risk. A significant negative correlation between the association results of APOE-ε4 and APOE-ε2 for metabolic health traits (Kendall’s τ = −0.4; *P* = 5.3 × 10^−6^) was observed, suggesting the antagonistic effect of the APOE alleles on the metabolism of fats in the body. Apart from this expected result, we also observed a significant negative correlation between APOE-ε2 and familial risk among blood cell traits (*P* = 2.9 × 10^−6^). There were also negative correlations of borderline significance between APOE-ε4 and APOE-ε2 for blood biochemistry, and between APOE-ε4 and familial risk for metabolic health traits. There were no significant correlations between polygenic and familial risk in any of the phenotypic categories, despite both constituting proxies of genome-wide (non-APOE) genetic risk for AD. We repeated the analyses using only those individuals with both PRS and familial risk data (*n* = 286,427), and re-examined the correlation between the two (Supplementary Fig. 3). Again, no significant correlations were observed between the two and there was minimal difference in the results.

### Trait associations with APOE-ε4 and APOE-ε2

The strongest associations between any of the AD genetic risk factors and the 273 traits tested are between the APOE-ε4 and APOE-ε2 dosages and the blood lipid levels (Table 1). APOE-ε4 dosage is highly significantly associated with higher levels of “bad cholesterol”, including APO-B, LDL, total cholesterol, and triglycerides, and lower levels of “good cholesterol”, including APO-A and HDL. Consistent with this, APOE-ε4 carriers are more likely to take cholesterol-lowering medication. The reverse trends are observed for APOE-ε2 dosage, which is associated with significantly lower “bad cholesterol” and higher “good cholesterol” traits. The impact that APOE-ε2 dosage has on these cholesterol levels is striking, explaining ~9% overall variance in APO-B, ~6% variance in LDL and ~4% variance in total cholesterol, despite the ε2 allele being of low frequency (allele frequency 6.8% in the UK Biobank). Notable exceptions to these opposite trends in association between APOE-ε4 and APOE-ε2 and the lipid traits are observed for triglycerides, in which there is a highly significant positive association with APOE-ε4 and APOE-ε2, and decreased levels of lipoprotein A with APOE-ε4 and APOE-ε2 dosage. Given that APOE-ε4 increases the risk of AD, while APOE-2 reduces AD risk, these results suggest that triglycerides and lipoprotein A may not play a direct role in AD pathology.

As expected, APOE-ε4 is associated with an increased risk of cognitive impairments (Fig. 1, Supplementary Table 1). APOE-ε4 carriers show cognitive decline across several cognitive tests, for example, taking longer to complete the Trail Marking Test and making fewer correct matches in the Digit symbol substitution test. No significant associations were observed between APOE-ε2 and cognitive function, which may reflect a smaller AD protective effect offered by the ε2 allele relative to the AD risk of the ε4 allele [30]. An example of a trait that APOE-ε4 and APOE-ε2 have effects on that are particularly consistent with their effects on AD is alanine aminotransferase (APOE-ε4 effect = −0.04; APOE-ε2 effect = 0.018; see Supplementary Table 1), which adds some support to its recently reported association with AD [31].

In addition to its effect on blood lipids, APOE-ε2 dosage is strongly associated with reduced levels of reticulocyte in blood, including immature reticulocyte fraction, high light scatter reticulocyte percentage, high light scatter reticulocyte count, reticulocyte percentage, and reticulocyte count (see Table 1). APOE-ε4 is associated with reduced immature reticulocyte fraction, but not with the other reticulocyte-related traits.

Furthermore, APOE-ε4 dosage is strongly associated with lower levels of C-reactive protein (CRP), a lower incidence of diabetes and reduced regular use of aspirin. While the associations with diabetes and aspirin are consistent with the lower level of CRP found in APOE-ε4 carriers, their effects are unexpected in that diabetes [32, 33] and high levels of CRP [34, 35] have been reported as associated with an elevated risk of neurodegenerative diseases. The opposite directions of effect are observed in relation to APOE-ε2 carriers, who have higher levels of CRP and higher incidence of diabetes, but only a nominal increase in aspirin use.

APOE-ε4 is also a known risk factor for CVD [36]. To replicate this finding, we examined the effect of APOE on CVD in the UK Biobank using logistic regression, adjusting for the standard covariates and parental age. We observed a significantly higher risk of heart attack in APOE-ε4 carriers, but no associations were observed for angina, stroke and high blood pressure. Conversely, the APOE-ε2 allele appears to decrease the risk of heart attack and angina, but not in stroke or high blood pressure.

**Fig. 3:**
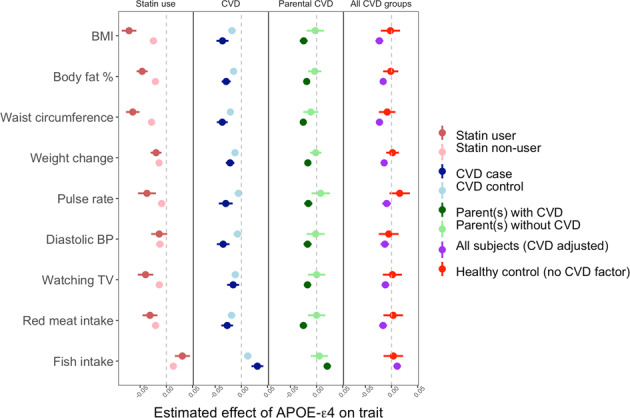
Differential effect of APOE-ε4 on selected health traits according to cardiovascular disease (CVD) status. All the models are adjusted for the standard covariates including age, sex, age^2^, age*sex interaction term, socioeconomic status based on Townsend deprivation index, genotyping batch, assessment centre, and the first 12 principal components of ancestry as covariates. In the model of all subjects (CVD adjusted), indicated by red colour, 3 CVD factors including statin use, CAD and parental CAD and lifestyle score are additionally adjusted. There are 53,173 statin users and 270,745 statin non-users in the data, 94,170 CVD cases and 229,104 CVD controls, and 232,286 participants with a parent with CVD and 46,914 with parents without CVD.

In our initial analyses we observed multiple examples of the different forms of genetic risk for AD associated with positive health outcomes, including healthier dietary intake, lower BMI, increased lung function and even increased height. These effects were also observed for APOE-ε4 dosage. To investigate these unexpected results, we re-evaluated the APOE-ε4 associations with metabolic traits, stratifying individuals according to their CVD status in terms of (i) statin use, (ii) self-reported CVD condition, and (iii) family history of CVD. Overall, there was a marked reduction in the APOE-ε4 effect on these factors among those individuals who were ‘controls’ for CVD (Fig. 3). For example, APOE-ε4 dosage is strongly associated with decreased BMI in statin users but this effect is largely ameliorated (two-sided *t*-test *P*-value = 3.1 × 10^−9^) in statin non-users, while APOE-ε4 dosage is associated with reduced red meat intake in individuals with a parent with CVD (*P* = 1.5 × 10^−11^) but not in those with no parent with CVD (*P* = 0.99). Possible explanations for these results are (1) collider bias caused by stratifying by CVD risk, (2) positive lifestyle changes made by APOE-ε4 carriers who are made aware of their increased CVD risk due to themselves, or their parent, being diagnosed with CVD or being prescribed statins. These alternatives warrant further study.

### Trait associations with polygenic risk

Polygenic risk, captured here by the AD PRS, is associated (*P* < 4.5 × 10^−5^) with 16 traits across blood biochemistry, blood cell traits, metabolic health and general health (Fig. 1, Table 1, Supplementary Table 1). Despite the highly significant associations between the APOE alleles and the lipid traits, the AD PRS shows no evidence of association with the lipids. This indicates that if lipid metabolism plays an important role in AD pathology, then this is largely restricted to individuals with APOE-ε4 rather than with high genetic risk according to present AD PRS, albeit current AD PRS (without APOE) explain only a tiny fraction of variance in AD (~1%). The only traits showing some evidence for shared effects across APOE and polygenic risk are several reticulocyte measures, including reticulocyte percentage and count, Aspartate aminotransferase and diabetes. Individuals with higher AD PRS are less likely to start insulin treatment within the first year of diagnosis of diabetes. Although the higher prevalence of insulin abnormalities and insulin resistance are reported in patients with AD [37, 38], how AD genetic risk impacts the clinical profiles for diabetic patients, leading to reduced or delayed initialization of insulin therapy determined by their physicians, is unclear.

AD PRS is associated with higher standing height, higher calcium levels and improved lung function, as measured by increased forced expiratory volume in one second (FEV1). This is in conflict with some previous epidemiological studies. Increased adult height has reported associations with better cognitive performance and reduced risk of AD [39–41], which may relate to increased cognitive reserve associated with the larger head circumference or better nutrition leading to greater height and reduced AD risk [39]. Moreover, individuals with impaired lung function have been reported to have a higher risk of cognitive decline and dementia [42, 43]. One potential explanation for these unexpected findings is that the AD PRS may include genetic effects associated with higher longevity if the AD studies used in the latest AD GWAS [9] included older cases than controls. While most AD studies age-match cases and controls, not all are able to, which allows the possibility of a systematic difference in longevity between cases and controls. To account for the longevity effect—a possible confounder in our results of AD PRS—we repeated the analyses adjusting for longevity PRS [44] as a covariate. However, the difference in the results was minimal (Supplementary Fig. 4), which may be due to the low predictive power of the present longevity PRS (*R*^2^ of 0.19%).

In terms of metabolic health, individuals with higher AD PRS are associated with more lean body mass, having higher basal (resting) metabolic rate, arm fat-free mass, trunk fat-free mass and trunk predicted mass. In contrast, APOE-ε4 carriers are associated with lower levels of lean body mass. However, the trait variance explained by the PRS associations is extremely small and so should be considered with caution.

### Trait associations with familial risk

Familial risk for AD, as defined by number of parents with AD, controlled for parental age, is significantly associated (*P* < 4.5 × 10^−5^) with 27 of the 273 traits. The profile of traits that familial risk and the other forms of AD risk are associated with is markedly different (Fig. 1, Table 1). Familial risk is mainly associated with psychosocial factors, which appear to be directly linked with caring for a parent with Alzheimer’s and/or anxiety over suffering from AD themselves in the future. For example, the strongest association is with experience of illness, injury, bereavement, the stress in the last 2 years (*P* = 1.4 × 10^−55^), followed by higher neuroticism score, increased tiredness in last two weeks, increased depressed mood in last two weeks, seen doctor for nerves or depression and reduced family relationship satisfaction. In addition, individuals who have or had parents with AD report a higher number of non-cancer illnesses. However, given the broad definition of non-cancer illness, which includes cardiovascular, respiratory and neurological disease, it is not possible to determine whether this is likely a consequence of the negative impact on their psychosocial health or due to other effects of having increased risk of AD.

The absence of associations between familial risk and the traits that the other forms of AD risk are associated with is likely a combination of the low convergence of effects between APOE effects and AD risk factors genome-wide, indicated by the results of AD PRS, and the greater heterogeneity of familial risk of AD, which incorporates rare genetic and all environmental factors.

**Fig. 4:**
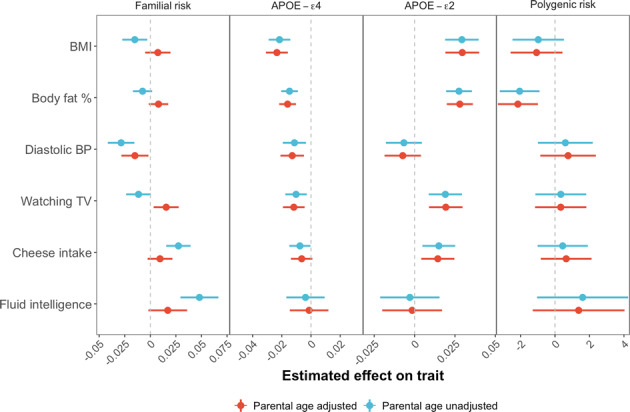
Effect of parental age adjustment in different forms of AD genetic risk. Estimated effects (standardized) of familial risk, APOE-ε4, APOE-ε2, polygenic risk on selected traits using the primary analysis model (Fig. 1, see Methods), with or without adjustment of parental age. Points show effect size estimates and whiskers show 95% confidence intervals.

Accounting for the survivor effect is critical when using familial risk as a predictor. In an initial analysis that we performed without controlling for parental age, we observed that individuals with positive AD family history appeared to be healthier or of higher socio-economic position across a range of traits, including having significantly lower diastolic blood pressure, lower TV watching and higher fluid intelligence. Individuals with LOAD generally have higher longevity than random members of the population, given the late onset of the disease. Thus, individuals whose parents have LOAD on average have parents with relatively high longevity, which is associated with a favourable genetic profile, socioeconomic advantage, and better health factors, all of which are inherited by offspring through a combination of the environment and genetics. However, once parental age is accounted for as a covariate then these associations are ameliorated or eliminated (Fig. 4); for example, diastolic blood pressure (Unadjusted: *P* = 1.7 × 10^−5^; Adjusted: *P* = 0.026), and fluid intelligence score (Unadjusted: *P* = 3.9 × 10^−7^; Adjusted: *P* = 0.078). The effects of APOE-ε4, APOE-ε2 and polygenic risk remain unaffected by the parental age adjustment.

### Investigation of interaction with age

Finally, we investigated how the effects of AD genetic risk factors on traits varied across age. We also investigated age effects stratified by sex and tested for sex differences (see Methods). These are not individual-level longitudinal analyses, since all participants only provide data at a single time-point, and so the results may be influenced by generational effects. However, if generational effects on a trait are small relative to age effects, then these results should provide a good proxy to longitudinal analyses.

**Fig. 5:**
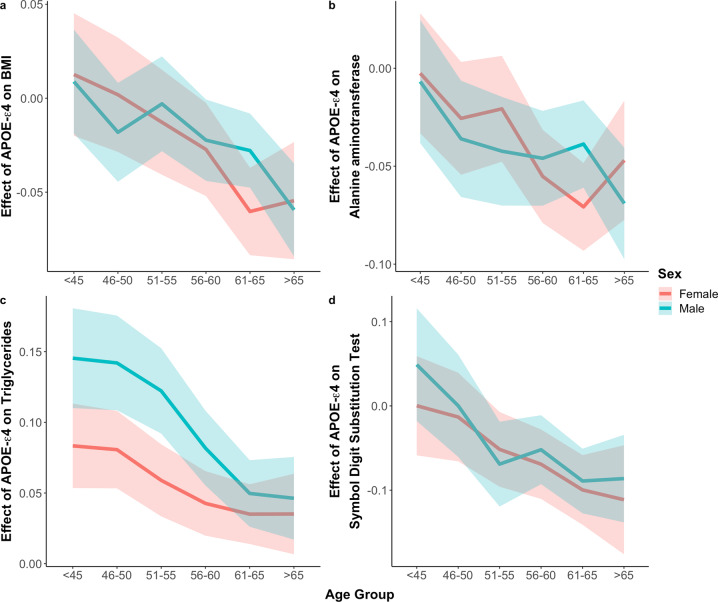
Effect of APOE-ε4 across different age groups on BMI, Alanine aminotransferase, Triglycerides and the Symbol Digit Substitution Test. Analyses are performed stratified by sex. The shaded area corresponds to the 95% confidence interval. Note that the analysis described in the text involves testing for an APOE-ε4*age interaction explicitly, with age considered as a continuous variable; the effect size of APOE-ε4 is estimated in age groups here for visualization of the effect.

Regression results using the sex-combined sample show a significant interaction (*P* < 8 × 10^−5^; see Methods) between the APOE-ε4 effect and age for 22 traits, including 4 blood lipid traits, Urate, Alanine aminotransferase, 15 metabolic traits relating to adiposity, and the Symbol Digit Substitution Test (Fig. 5**;** Supplementary Table 2). Assuming that there are not strong generational effects, then individuals with APOE-ε4 appear to have increasingly lower body fat across age (BMI: interaction *P* = 4.4 × 10^−10^; Body fat %: interaction *P* = 2.6 × 10^−8^), increasingly lower alanine aminotransferase (interaction *P* = 8.3 × 10^−6^) and increasing cognitive decline as measured by the Symbol Digit Substitution Test (interaction *P* = 9.7 × 10^−8^). These data indicate that cognition function can start to deteriorate from age 40 to 50 under the influence of APOE-ε4. This analysis, therefore, highlights, in addition to cognitive decline, adiposity and alanine aminotransferase as potentially having close links with AD pathology given the timing of the effect of APOE-ε4 on them. This adds evidence to a longitudinal study that reported an increasing negative association of APOE-ε4 with BMI across age in a prospectively measured cohort [45], as well as a recent study that implicated alanine aminotransferase in AD diagnosis and pathophysiology [31].

APOE-ε2, which is associated with reduced LDL and cholesterol during the ages 40–50 in these data, is associated with increasing LDL and cholesterol with age (LDL: interaction *P* = 3.8 × 10^−6^; Cholesterol: interaction *P* = 1.1 × 10^−5^). It should be noted that the interactions with age observed here in relation to the APOE variants may reflect the survivor effect (discussed in the previous section). For example, the elevated risk of CVD among APOE-ε4 carriers may be sufficient that they need to have markedly better metabolic health to survive mid-life.

No significant interaction effects were observed for AD PRS. Significant interactions between the effects of familial risk and age were observed for illness, injury, bereavement, the stress in the last 2 years, and frequency of depressed mood. Both factors had an apparently decreasing effect with age. Note that familial risk with AD is associated with these stress/mood factors in all age groups, but with a reducing effect across age, which may reflect a greater burden for relatively younger carers and for those with a younger parent with AD.

There were no significant differences in interaction effects between males and females for these analyses.

## Discussion

This study is the most powerful systematic comparison of how the major forms of AD genetic risk affect the phenome so far. We were able to make direct comparisons between the effects of forms of AD risk, such as APOE-ε2 and familial risk, which can only be powerfully studied together in exceptionally large cohorts.

The largest associations were between the APOE alleles and lipid metabolism. APOE-ε2 explains ~4%, ~6% and ~9% variance in cholesterol, LDL and Apolipoprotein-B (APO-B), respectively, while APOE-ε4 explains 1–2% variance in each. These are striking effects for single common variants. There is already substantial evidence for a role of lipid metabolism [46, 47], and its effects, such as atherosclerosis [48], on Alzheimer’s pathophysiology. However, there is no evidence in these data for an association between polygenic or familial risk and lipid traits. While the relatively low power of the polygenic and familial risk factors mean that convergence of effects on lipid metabolism and AD across a subset of genetic loci cannot be ruled out, it is clear that polygenic and familial risk should be separated out from APOE effects in AD research into lipid metabolism to avoid conflating their effects.

APOE-ε4 had its largest impact on any single trait on CRP—a marker of low-grade inflammation—explaining ~2% variance in CRP. However, as observed elsewhere [49], APOE-ε4 dosage is associated with reduced CRP, despite the fact that low-grade inflammation is an established risk factor for CVD [50, 51] and AD [34, 35]. This apparent paradox may be explained by the potentially divergent processes leading to AD from APOE-ε4 and from the genetic risk of AD across the rest of the genome.

We identified 98 traits significantly associated with at least one of the four forms of AD risk. While likely most of these traits are associated with AD genetic risk due to (horizontal) pleiotropic effects, the possibility that they may be mediators between AD genetic risk and AD can be considered broadly in terms of their convergence across different forms of AD, the consistency of APOE-ε4/APOE-ε2 trait and AD effects, and their changing effects with age. Traits highlighted due to several of these factors include: C reactive protein (CRP), BMI, diabetes, alanine aminotransferase and reticulocyte blood cell measures. For example, alanine aminotransferase was highlighted due to the fact that APOE-ε4 has a threefold higher, but opposing effect, on it as APOE-ε2, consistent with APOE effects on AD, and due to the significant interaction between the APOE-ε4 effect on it and age (Fig. 5). This adds further evidence to the reported associations between alanine aminotransferase and AD [31].

Despite some evidence of convergent effects on traits among different genetic risk factors for AD, the overall pattern was of contrasting profiles of associations between the APOE variants, polygenic risk, and familial risk. Much of this is likely caused by the relatively low power of the PRS and familial risk factors, and that genetic risk variants for any disease also often have pleiotropic effects on a range of other traits. However, part of this heterogeneity of effects on traits may reflect heterogeneity in the disease etiology itself, and so these findings warrant further interrogation. Some of the associations can be more easily explained, such as familial AD risk being associated with factors relating to caring for a parent with Alzheimer’s, but these are still important to account for in AD research and in the clinical care of Alzheimer’s patients. Our study also re-iterated the importance of accounting for the survivor effect in AD research, in relation to both the overlap between CVD and AD risk factors (namely APOE-ε4 here) and the fact that individuals with late-onset AD will typically have had an otherwise healthy profile given their longevity.

All of the associations reported in this study were investigated univariately in terms of traits, are based on cross-sectional observational data, and are analysed in a large sample with the power to detect very small but potentially unimportant effects, and so all results should be considered exploratory in nature.

We expect our study to act as a useful resource to aid in understanding the effects that different forms of AD genetic risk have on the phenome. We hope that our results provide an ideal starting point for further observational interrogation of AD aetiology, including replicating these analyses in other populations, and that insights gained here provide leads for experimental studies that may be able to shed light on those causal mechanisms that both underlie these associations and lead to AD.

## Supplementary information

Supplementary Material

Supplementary Table

## Data Availability

Analytic code to define outcomes and regression models for this work is available upon request.
