# Supplementary material for: Heterogeneous effects of genetic risk for Alzheimer’s disease on the phenome
Source: Transl Psychiatry. 2021 Jul 23;11:406. doi: 10.1038/s41398-021-01518-0 (PMC8302633; doi:10.1038/s41398-021-01518-0)
Supplement: Supplementary file 1 — Supplementary Material [file 41398_2021_1518_MOESM1_ESM.docx]

Supplementary Material

# Note 1: Data processing on phenotypic data in the UK Biobank

## Cognitive function

Participants were invited to complete cognitive function test online. Results from the Prospective Memory Test (prospective memory), Pair Matching Test (number of incorrect matches in round), Fluid Intelligence Test (fluid intelligence,), Reaction Time Test (reaction time), Symbol Digit Substitution (number symbol digit matches made correctly), Trail Making Testing A (Duration to complete numeric path) and B (Duration to complete alphanumeric path)were used.

F6138 was used to derived two variables: Attend college, and Qualification. For Attend college, we created a binary variable whether or not a participant attained a college or university degree. For Qualification, we reversed to the order of coding to make it more interpretable, with the college/university being the highest score ^1^.

## Metabolic health

**Dietary variables**: We followed the Bradbury et. al to construct the dietary variables: red and processed meat, red meat, processed meat, poultry, total fish, daily fish, cheese, fruit, vegetables, fibre score, alcohol, tea and coffee.

**Exercise-related**: (We extracted and derived the following variables: Walking exercise(F22037), Moderate exercise(F22038), Vigorous exercise(F22039), Time spent driving(F1090), Time spent using computer(F1080), Time spent watching TV(F1070)

**Sleep)**: We used Sleep duration(F1160) to derive two variables: Long sleep duration (>9 hours), short sleep duration (<7 hours). Morning/evening person (F1180), insomnia(F1200), snoring(F1210) were also included ^2^.

**Smoking:** Smoking status(F20116) was used.

**Healthy Lifestyle Score :** We followed Louirda al et. ^3^ to construct healthy lifestyle score based on smoking status, physical activity, diet and alcohol consumption.

**Impedance:** For the following variables, we used the measurement for the right side: Arm fat free mass (F23121), Arm fat mass (F23120), Arm fat percentage (F23119), Arm predicted mass (F23122), Impedance of arm (F23109), Impedance of leg (F23107), Leg fat free mass (F23113), Leg fat mass (F23112), Leg fat percentage (F23111), Leg predicted mass (F23114). The remaining variables in this category includes: Basal metabolic rate (F23105), Body fat percentage (F23099), BMI (F23104), Impedance of whole body (F23106), Trunk fat free mass (F23129), Trunk fat mass (F23128), Trunk fat percentage (F23127), Trunk predicted mass (F23130), Weight (F23098), Whole body fat free mass (F23101), Whole body fat mass (F23100), and Whole body water mass (F23102).

**Physical measure**: Anthropometry including Hip circumference (F49), Waist circumference (F48) are included.

## Psychosocial health

All variables were extracted and derived from Category 100060. We excluded the 12 variables (F1920 to F2030) that make up the neuroticism score to avoid redundant information.

## General Health

Under Health and medical history (Category 100036), variables were extracted and derived from General health (Category 100042), Pain (Category 100028), Operations (Category 100047), Medical condition (Category 100044), Medication (Category 100045). In addition, we also extracted health-related variables under Verbal interview (Category 100071) including variables from Birth weight (F20022), Number of operations self-reported (F20004), Number of self-reported cancers (F134), Number of self-reported non-cancer illness (F135), Number of operations (F136) and Number of treatment medication taken (F137).

Automated reading from diastolic blood pressure (F4079), systolic blood pressure (F4080), pulse rate (F102) were used. Best measures of Forced vital capacity (F20151), Forced expiratory volume in 1-second (F20150), Hand grip strength (right) (F47) were included in our analyses. Seated height (F51), Sitting height (F20015), Standing height (F50) were also included.

## Blood biochemistry

For traits under Blood biochemistry, we followed the Sinnott-Armstrong et al. ^4^ to identify 4,608 participants who did not take statin at baseline (2006-2010) but were taking statin at the first repeat assessment (2012-2013). We then derived the statin correction factor and apply the correction to all individuals who were already taking statin at baseline. We only applied this statin adjustment to traits with significant difference in Wilcox signed rank test for paired sample. A total of 29 blood biochemical markers were included.

## Blood cell traits

We also extracted blood cell traits from Blood Count, We followed the QC method from Astle et al. ^5^ study to process the data. A total of 32 blood cell traits are included.

**Supplementary Figure 1. Associations between polygenic risk (PRS) of AD, Parkinson’s Disease (PD), Major Depressive Disorder (MDD), Diabetes and Height and 273 traits in the UK Biobank.** As in our main analyses on AD, we removed “cases” in each of the corresponding disease outcomes, based on ICD-10 code and self-report, so that analyses are performed in the general population sample. Polygenic risk scores were constructed using the latest available GWAS of AD^7,8^ (see **Methods**), Parkinson’s Disease (PD)^9^, Major Depressive Disorder (MDD) (no UK Biobank to avoid sample overlap)^10^, Type II Diabetes^11^ and height^12^ as base data, applying the previously reported best *P*-value threshold for PRS calculation^13,10,14^ (if unavailable, we applied best *P*-value threshold from training the PRS using clinical cases in the UK Biobank). Consistent with our main analysis, we removed the “cases” in each of the corresponding disease trait. The grey dotted lines correspond to the *P*-value significance threshold (*P <* 4.5x10^-5^). The Y-axis has been truncated to show all associated traits to improve visualization, with extreme results shown in the grey area with inverted triangles.

| AD PRS  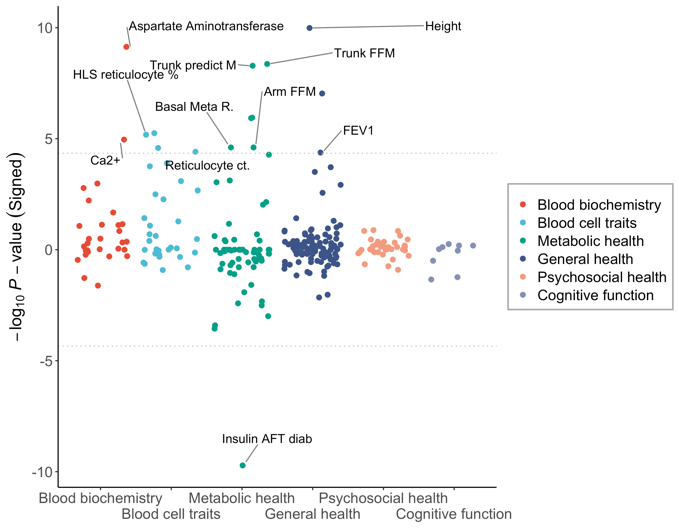 | PD PRS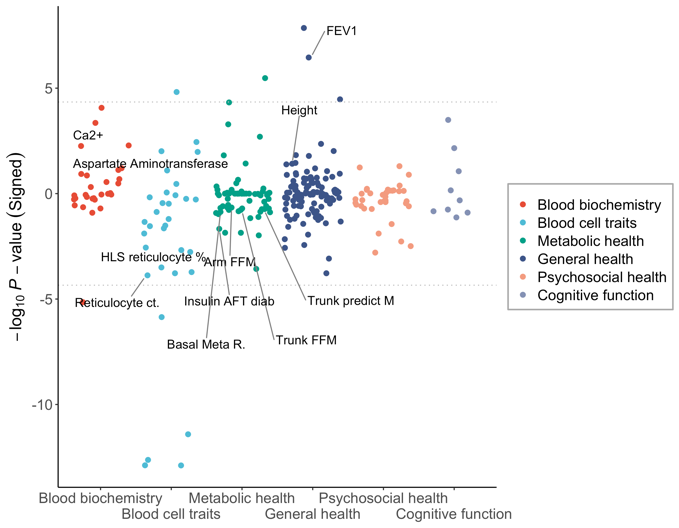 |
| --- | --- |
| MDD PRS  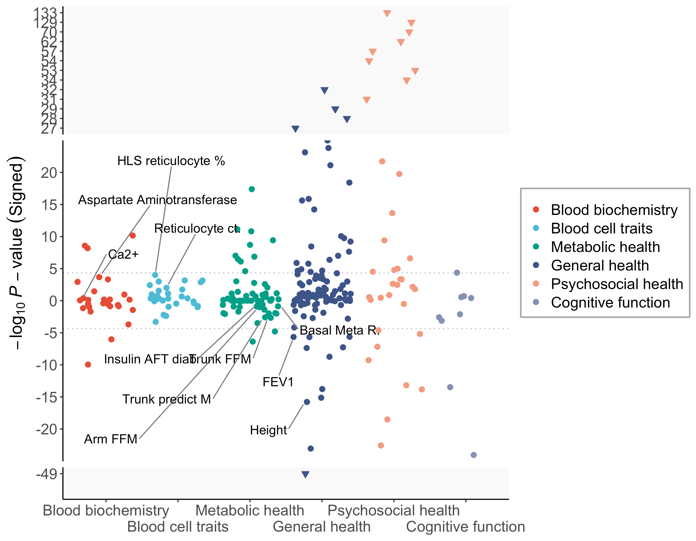 | Type II Diabetes PRS  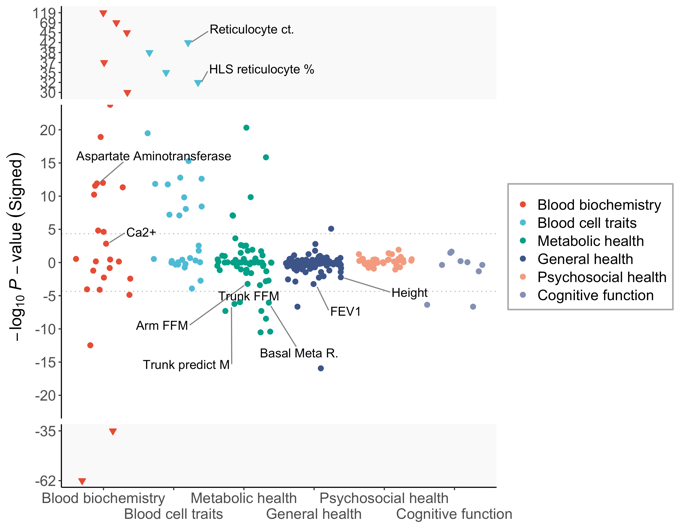 |
| Height  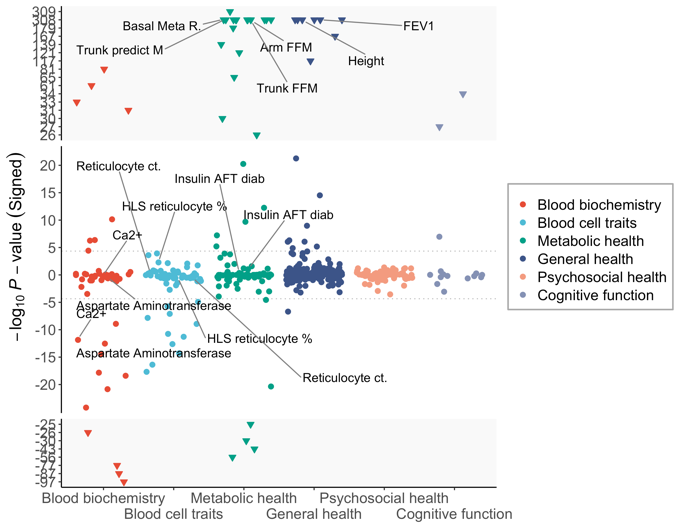 |  |

**Supplementary Figure 2. Associations between familial risk of AD, Parkinson’s Disease (PD), Major Depressive Disorder (MDD) and Diabetes and 273 traits in the UK Biobank.** Self-reported family history in parents (F20110 and F20107) was used (the question relating to diabetes does not specify type I or type II). Subjects with no parent affected with corresponding disease is coded as 0, with one affected parent as 1, and with two affected parents as 2. In addition, we removed the “cases” in each of the corresponding disease trait. The grey dotted lines correspond to the *P*-value significance threshold (*P <* 4.5x10^-5^). The Y-axis has been truncated to show all associated traits to improve visualization, with extreme results shown in the grey area with inverted triangles.

| AD familial risk  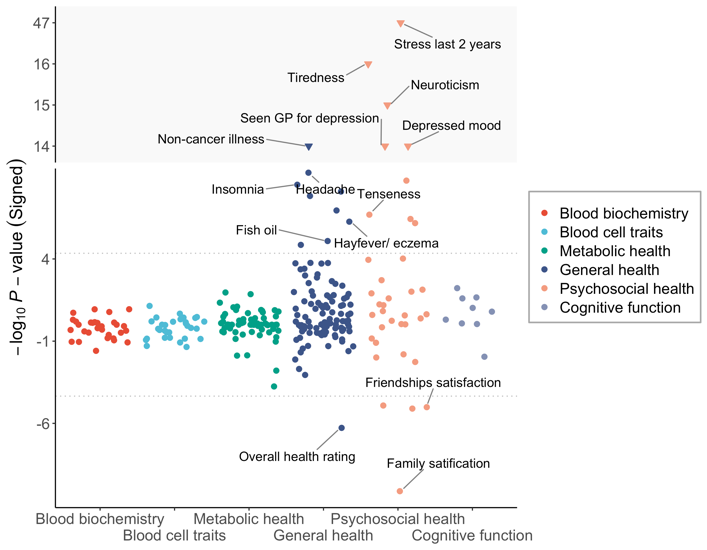 | PD familial risk  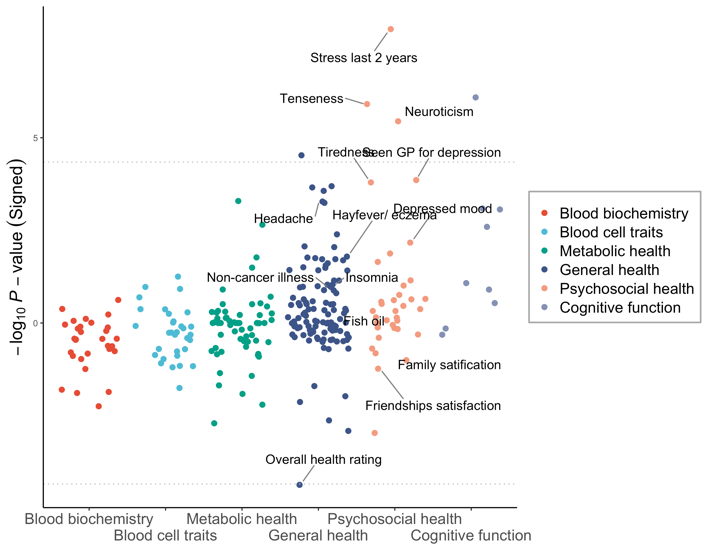 |
| --- | --- |
| MDD familial risk  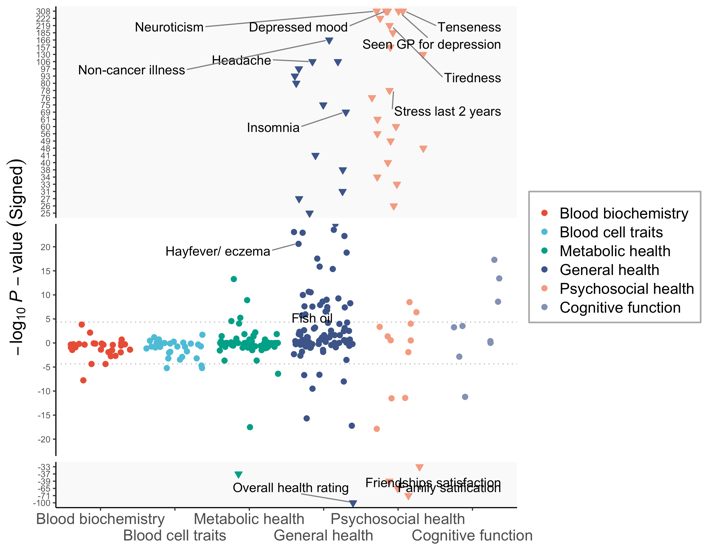 | Diabetes familial risk  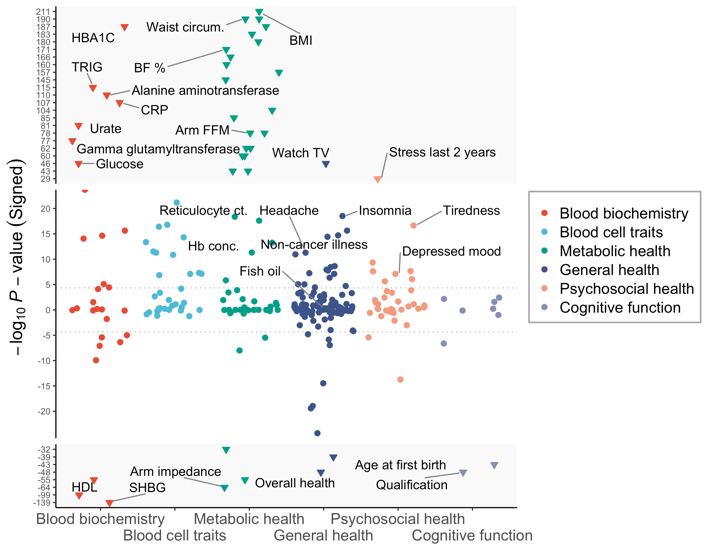 |

**Supplementary Figure 3**. **Correlations between the trait association results of different forms of AD genetic risk in overlapped sample.** Different to Fig 2, the analyses of polygenic and familial risk were restricted to sample (n=286,426) who contains information on both parental history of AD and polygenic score.


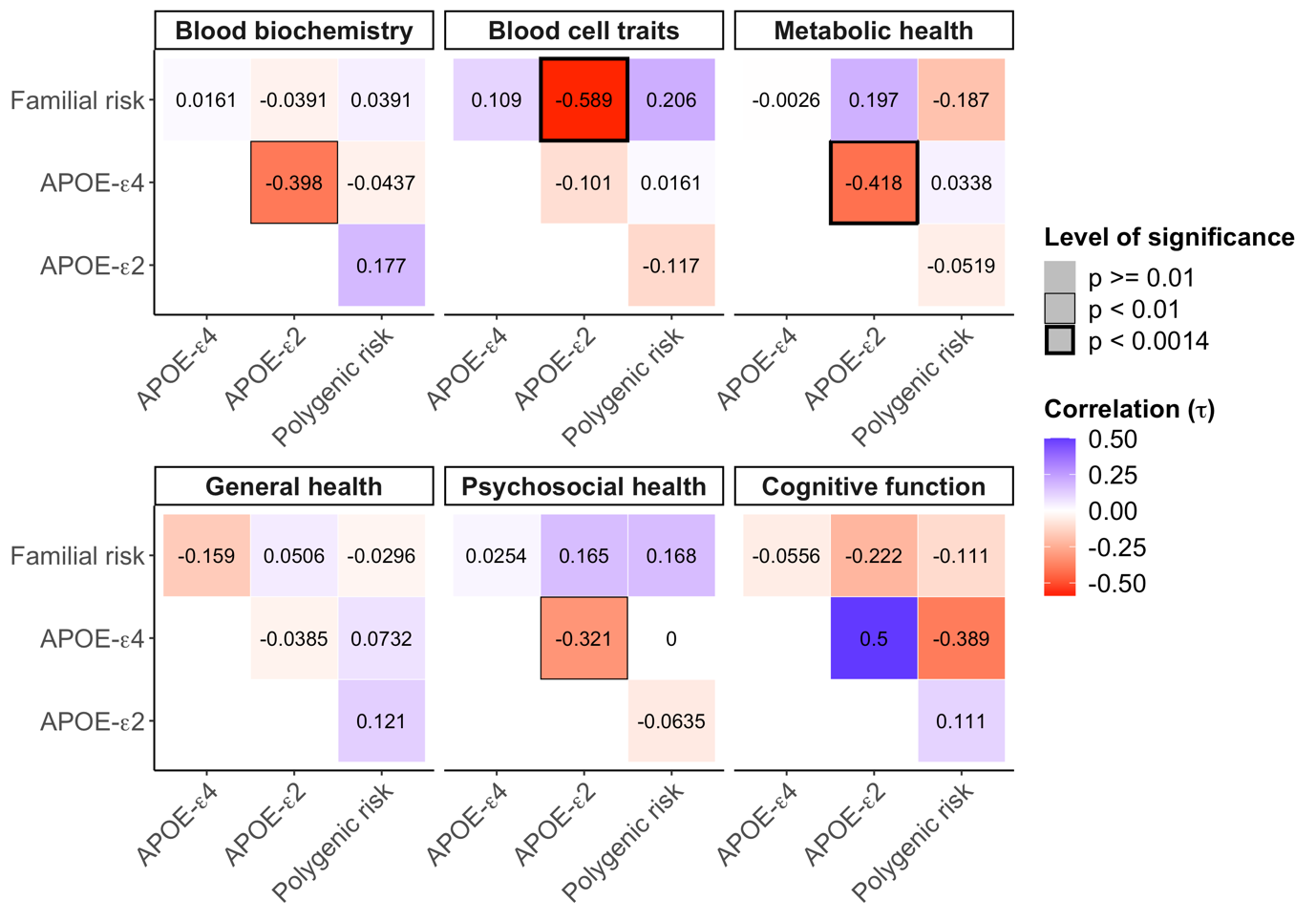


**Supplementary Figure 4. Associations between polygenic risk (AD PRS) and 273 traits in the UK Biobank, with additional adjustment for longevity PRS.** Since the UKB sample were at relatively young ages (40-69 years old), we used the parental age information to define parental longevity, as a proxy for longevity effect. Following the study of Deelen et al. ^6^, cases were participants with at least one parent aged above 90^th^ percentile and who were still alive themselves at recruitment, while controls were participants for whom both parents had died at or before the age of the 60^th^ percentile. PRS for longevity was constructed using the summary statistics of the longevity GWAS ^6^, and controlled for in the regression models as one of the covariates.


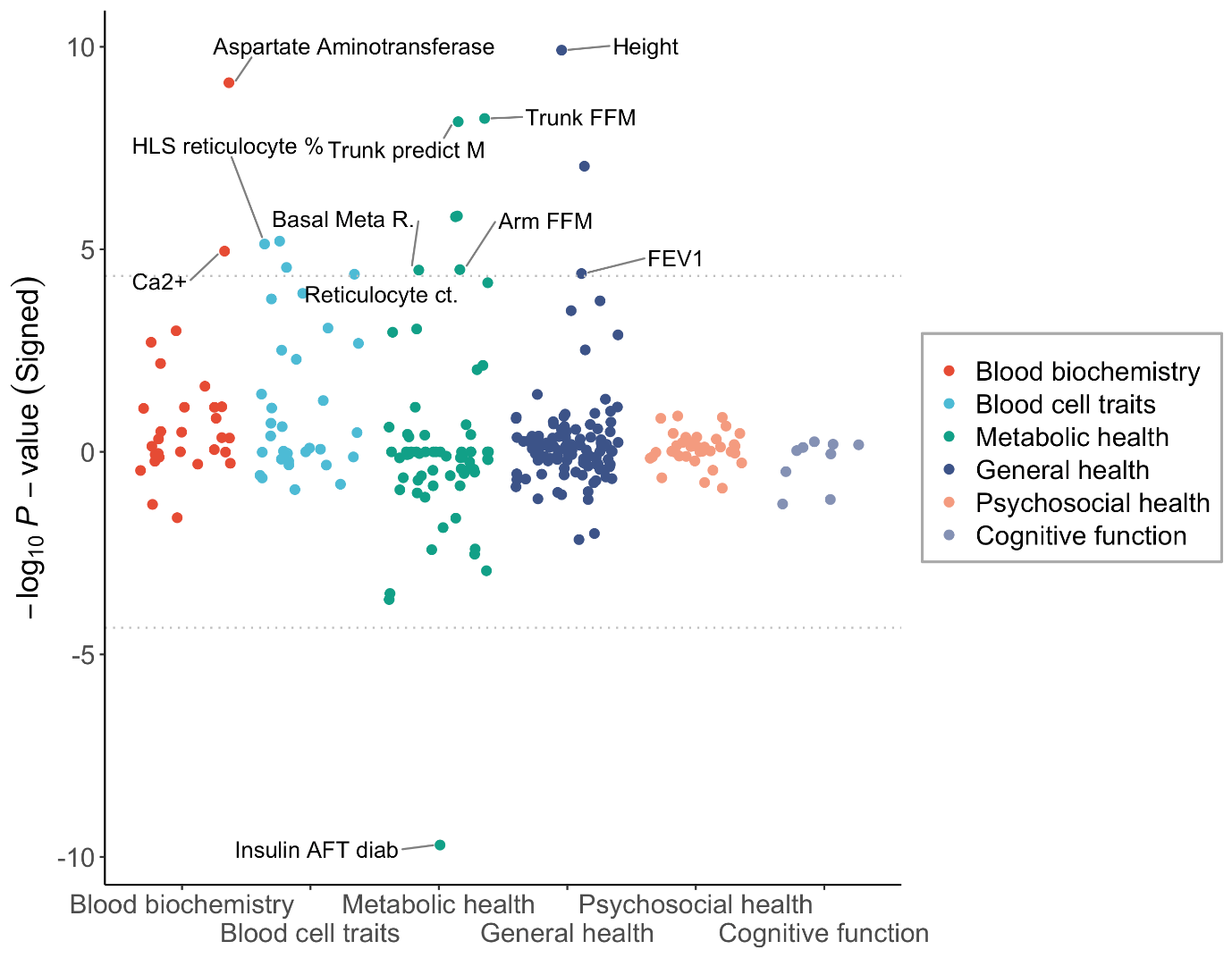


Reference

1. Escott-Price, V. *et al.* Genetic liability to schizophrenia is negatively associated with educational attainment in UK Biobank. *Molecular Psychiatry* **25**, 703–705 (2020).

2. Kyle, S. D. *et al.* Sleep and cognitive performance: Cross-sectional associations from the UK Biobank. *Sleep Med* **38**, 85–91 (2017).

3. Lourida, I. *et al.* Association of Lifestyle and Genetic Risk With Incidence of Dementia. *JAMA* **322**, 430–437 (2019).

4. Sinnott-Armstrong, N. *et al.* Genetics of 38 blood and urine biomarkers in the UK Biobank. *bioRxiv* 660506 (2019) doi:10.1101/660506.

5. Astle, W. J. *et al.* The Allelic Landscape of Human Blood Cell Trait Variation and Links to Common Complex Disease. *Cell* **167**, 1415-1429.e19 (2016).

6. Deelen, J. *et al.* A meta-analysis of genome-wide association studies identifies multiple longevity genes. *Nat Commun* **10**, 3669 (2019).

7. Kunkle, B. W. *et al.* Genetic meta-analysis of diagnosed Alzheimer’s disease identifies new risk loci and implicates Aβ, tau, immunity and lipid processing. *Nat Genet* **51**, 414–430 (2019).

8. Lambert, J. C. *et al.* Meta-analysis of 74,046 individuals identifies 11 new susceptibility loci for Alzheimer’s disease. *Nat Genet* **45**, 1452–1458 (2013).

9. Nalls, M. A. *et al.* Identification of novel risk loci, causal insights, and heritable risk for Parkinson’s disease: a meta-analysis of genome-wide association studies. *Lancet Neurol* **18**, 1091–1102 (2019).

10. Wray, N. R. *et al.* Genome-wide association analyses identify 44 risk variants and refine the genetic architecture of major depression. *Nature Genetics* **50**, 668–681 (2018).

11. Scott, R. A. *et al.* An Expanded Genome-Wide Association Study of Type 2 Diabetes in Europeans. *Diabetes* **66**, 2888–2902 (2017).

12. Wood, A. R. *et al.* Defining the role of common variation in the genomic and biological architecture of adult human height. *Nat Genet* **46**, 1173–1186 (2014).

13. Ibanez, L. *et al.* Parkinson disease polygenic risk score is associated with Parkinson disease status and age at onset but not with alpha-synuclein cerebrospinal fluid levels. *BMC Neurol* **17**, (2017).

14. Liu, W., Zhuang, Z., Wang, W., Huang, T. & Liu, Z. An Improved Genome-Wide Polygenic Score Model for Predicting the Risk of Type 2 Diabetes. *Front. Genet.* **12**, (2021).
